# The Impact of Pre-sleep Protein Ingestion on the Skeletal Muscle Adaptive Response to Exercise in Humans: An Update

**DOI:** 10.3389/fnut.2019.00017

**Published:** 2019-03-06

**Authors:** Tim Snijders, Jorn Trommelen, Imre W. K. Kouw, Andrew M. Holwerda, Lex B. Verdijk, Luc J. C. van Loon

**Affiliations:** Department of Human Biology, NUTRIM School of Nutrition and Translational Research in Metabolism, Maastricht University Medical Centre (MUMC+), Maastricht, Netherlands

**Keywords:** protein, exercise, satiety, amino acids, aging

## Abstract

This review provides an update on recent research assessing the effect of pre-sleep protein ingestion on muscle protein synthesis rates during overnight sleep and the skeletal muscle adaptive response to exercise training. Protein ingested prior to sleep is effectively digested and absorbed during overnight sleep, thereby increasing overnight muscle protein synthesis rates. Protein consumption prior to sleep does not appear to reduce appetite during breakfast the following day and does not change resting energy expenditure. When applied over a prolonged period of resistance-type exercise training, pre-sleep protein supplementation has a beneficial effect on the increase in muscle mass and strength. Protein ingestion before sleep is hypothesized to represent an effective nutritional strategy to preserve muscle mass in the elderly, especially when combined with physical activity or muscle contraction by means of neuromuscular electrical stimulation. In conclusion, protein ingestion prior to sleep is an effective interventional strategy to increase muscle protein synthesis rates during overnight sleep and can be applied to support the skeletal muscle adaptive response to resistance-type exercise training.

## Introduction

Resistance-type exercise training represents a potent stimulus to increase skeletal muscle mass and strength [see (1) for an extensive review]. Muscle protein synthesis as well as breakdown rates, are effectively stimulated following a single session of resistance type exercise, albeit breakdown rates are stimulated to a lesser extent (2, 3). However, in the absence of protein intake, the net muscle protein balance will remain negative (2, 3). Dietary protein intake shortly after exercise inhibits exercise-induced muscle protein breakdown and further augments the exercise-induced increase in muscle protein synthesis rate, resulting in a (more) positive post-exercise muscle protein balance. This synergistic effect between nutrition and exercise on the post-exercise muscle protein synthetic response has been well-established and forms an essential principle to enable the skeletal muscle adaptive response to more prolonged exercise training (4, 5). The muscle protein synthetic response following exercise has been observed to be modifiable to the type, amount, distribution, and timing of protein ingestion [see (6–8) for extensive reviews]. Recently, the concept of protein ingestion prior to sleep has been introduced as an additional meal moment to increase daily protein intake and increase overnight protein balance, which could further maximize the skeletal muscle adaptive response. In this review we will provide an update on the recent research investigating the impact of pre-sleep protein ingestion with and without exercise (training) on the muscle protein synthetic response in both young and older individuals.

## Pre-Sleep Protein Digestion and Absorption

**Figure 1:**
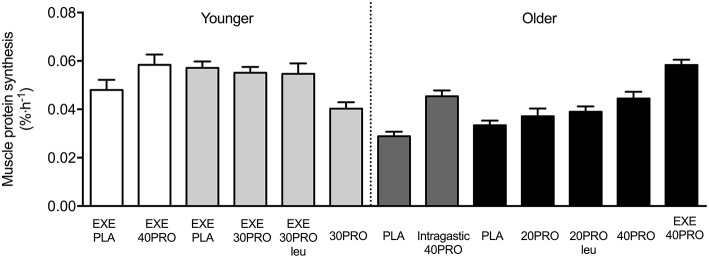
Overview of previous published overnight muscle protein synthesis studies at Maastricht University. EXE, single bout of resistance exercise; PLA, placebo; PRO, protein; Intragastic, Protein ingestion via intragastric tube; Leu, enriched with free crystalline leucine. White [Res et al. (11)] and light gray [Trommelen et al. (14, 18)] bars indicate study results from healthy young men. Dark gray [Groen et al. (10)] and black [Kouw et al. (17) and Holwerda et al. (13)] bars indicate study results from healthy older men.

In recent years, food ingestion prior to sleep has received considerable media attention. It has previously been assumed that food intake should be limited or avoided in the hours close to night-time sleep as it would have a negative impact on body composition and overall health, increasing the risk for cardiovascular diseases, such as obesity and diabetes [see also (9) for an extensive review]. Although this may be true when food is ingested in large quantities at night, more recent studies investigating the impact of smaller and single macronutrient (i.e., protein) foods have demonstrated positive physiological outcomes in humans. In addition, the benefits of night-time supply of nutrients for overnight recovery have been suggested to support muscle reconditioning and improve physical performance in athletes. Over the past decade, we have performed a considerable number of *in vivo* human studies to provide insight on the impact of pre-sleep protein ingestion on (post-exercise) overnight muscle protein synthesis in both young and older adults (10–18). Figure 1 provides a comprehensive overview of the studies performed in our laboratory, which will be discussed in more detail throughout this review.

In the study by Groen et al. (10) we demonstrated for the first time that administration of 40 g (intrinsically labeled) protein during sleep (via a nasogastric tube) is normally digested and absorbed in older adults, resulting in an increase in overnight muscle protein synthesis rates. This indicates that the gut functions appropriately at night and suggests that nocturnal protein administration may be applied as a nutritional strategy to increase muscle protein synthesis rates during overnight sleep. Although intragastric protein feeding during sleep may represent a feasible intervention strategy in certain clinical populations, it is far from being practical in a day-to-day situation in healthy individuals. In more recent studies by our laboratory, we have demonstrated that the ingestion of a single bolus of protein prior to sleep (ranging from 20 to 40 g protein) also leads to appropriate protein digestion and amino acid absorption during subsequent overnight sleep in both young (18) and healthy older adults (13, 16). In addition, our studies have shown no effect of pre-sleep protein ingestion on sleep onset latency, sleep quality, and/or next morning appetite in both young and older individuals (13, 18). As such, pre-sleep protein ingestion can be considered as an additional meal moment to increase total daily protein intake and improve overnight protein balance.

## Pre-Sleep Protein and Overnight Recovery

The concept of pre-sleep protein ingestion has been introduced as a way to increase overnight muscle protein synthesis rates when exercise is performed prior to bed-time. This is of particular relevance as previous research has shown that overnight muscle protein synthesis rates tend to be lower than those typically observed in the morning following an overnight fast (19). In the study by Res et al. (11) recreational athletes performed a single bout of resistance-type exercise in the evening. To maximize the immediate muscle protein synthetic response following exercise, all participants ingested 60 g of carbohydrates and 20 g of whey protein immediately after exercise. In addition, subjects were provided with either 40 g of casein protein or a placebo drink (water) immediately prior to sleep. Muscle protein synthesis rates were ~22% higher during overnight sleep when protein was consumed prior to sleep compared to participants ingesting the placebo drink (11). In contrast, in a more recent study from our laboratory we observed no significant increase in overnight myofibrillar protein synthesis rates in response to a single exercise session performed in the evening immediately followed by a post-exercise recovery drink (containing 20 g milk protein) and 30 g of casein protein with or without 2 g of crystalline leucine or placebo (water) prior to sleep (18). The apparent discrepancy between study outcomes may be explained by the more moderate amount of protein (30 g in the second study as opposed to 40 g protein in the first study) that was ingested prior to sleep. This may indicate that a dose-response relationship exists with pre-sleep protein ingestion, which is not in line with the immediate post-exercise recovery period (generally assessed over a relatively short 3–4 h recovery period as compared to a more prolonged 7.5 h overnight recovery period) during which 20 g has been reported to be sufficient to maximize muscle protein synthesis rates following a single bout of exercise in young healthy males (20, 21). Nevertheless, we have clearly shown that resistance-type exercise augments the overnight skeletal muscle adaptive response, with myofibrillar muscle protein synthesis rates being 37% higher when 30 g of pre-sleep protein (casein) ingestion is combined with a single session of resistance type exercise in the evening compared with pre-sleep protein ingestion only (14). Furthermore, the application of intrinsically labeled pre-sleep protein allowed us to demonstrate that 76% more of the pre-sleep protein derived amino acids were incorporated in myofibrillar protein when earlier that evening individuals engaged in a single resistance exercise session (14). Although the optimal dosage for pre-sleep protein ingestion remains to be determined, it has become clear that prior physical activity (e.g., resistance exercise) increases the efficiency by which pre-sleep protein derived amino acids are used in *de novo* muscle protein synthesis during overnight sleep.

Pre-sleep protein ingestion has been hypothesized to be a viable option to increase dietary protein intake to attenuate the loss of muscle mass with aging in older adults (5). In a recent study, we provided 40 g of casein protein prior to sleep in older adults and demonstrated and increase in overnight muscle protein synthesis rates (17). In this study, 40 g of casein ingested prior to sleep was compared with 20 g of casein with and without additional 1.5 g crystalline leucine, or a placebo. Ingestion of 20 g protein, as opposed to 40 g, did not result in a significant increase in overnight muscle protein synthesis rates when compared to the placebo condition. These results appear to be in line with studies performed during daytime demonstrating that the ingestion of a meal-like amount of protein (20 g) increases muscle protein synthesis rates by ~75% in healthy young individuals, whereas in an older population ~40 g protein is prerequisite to allow a similar rise in muscle protein synthesis rates during the postprandial period (17). Besides ingesting larger amounts of protein, fortifying lower amounts of protein with free leucine has been reported to further stimulate postprandial muscle protein synthesis rates in older adults (22–24). However, in the study by Kouw et al. (17), no differences in the post-prandial overnight muscle protein synthetic response were observed between 20 g casein protein ingested with or without additional free leucine (1.5 g). In this publication, we speculated that “*the absence of a stimulating effect after leucine co-ingestion could be attributed to the absence of a robust insulin response during the overnight period, the lack of sufficient amino acids as precursors for muscle protein synthesis during the relatively long overnight period, or simply that the stimulating properties of leucine are less evident during sleep”* (17). It is important to note that this study was performed in healthy older adults, whether more robust beneficial effects on overnight muscle protein synthesis can be observed when (small amounts of) protein is consumed prior to sleep by individuals with a relative low habitual dietary protein intake, like frail elderly, and/or more compromised clinical population, remains to be established. Furthermore, these studies were performed in the absence of performing physical activity or exercise in close proximity to the protein ingestion.

As already discussed, physical activity/exercise is known to further augment the postprandial rise in muscle protein synthesis in both young and older adults (25–27). In the study of Holwerda et al. (13) we showed that protein (40 g of casein) ingestion prior to sleep results in a positive overnight whole body protein balance in older adults. More importantly, overnight muscle protein synthesis rates were significantly higher when older adults performed a single exercise session earlier that evening. Overall, the existing studies suggest that pre-sleep protein consumption may represent an effective intervention to maintain muscle mass by increasing muscle protein synthesis rates during overnight sleep. In older and/or more clinically compromised populations the ingestion of a large bolus (40 g) of protein may not be feasible or practical, however, the study by Holwerda et al. (13) suggests that being physically active/performing exercise earlier that day may increase the beneficial effects when ingesting smaller amount of protein (<40 g) prior to sleep. During long periods of bed rest (e.g., due to illness or injury), however, physical activity can be severely restricted. The muscle protein synthetic response to protein ingestion is significantly reduced in response to physical inactivity (see (28) for extensive review). Neuromuscular electrical stimulation (NMES) represents an exercise mimetic that evokes muscle contractions and has been hypothesized to be an effective alternative to performing physical activity or exercise to attenuated disuse related anabolic resistance. Muscle protein synthesis rates have been reported to increase when NMES is performed in the morning after an overnight fast (29). More importantly, we have shown that NMES application prior to pre-sleep protein ingestion stimulates muscle protein synthesis during overnight sleep in healthy older adults (16). As such, the application of NMES with pre-sleep protein ingestion may represent a viable strategy to optimize overnight muscle protein balance and may be of significant clinical relevance to preserve skeletal muscle mass in bed-ridden, hospitalized patients.

**Figure 2:**
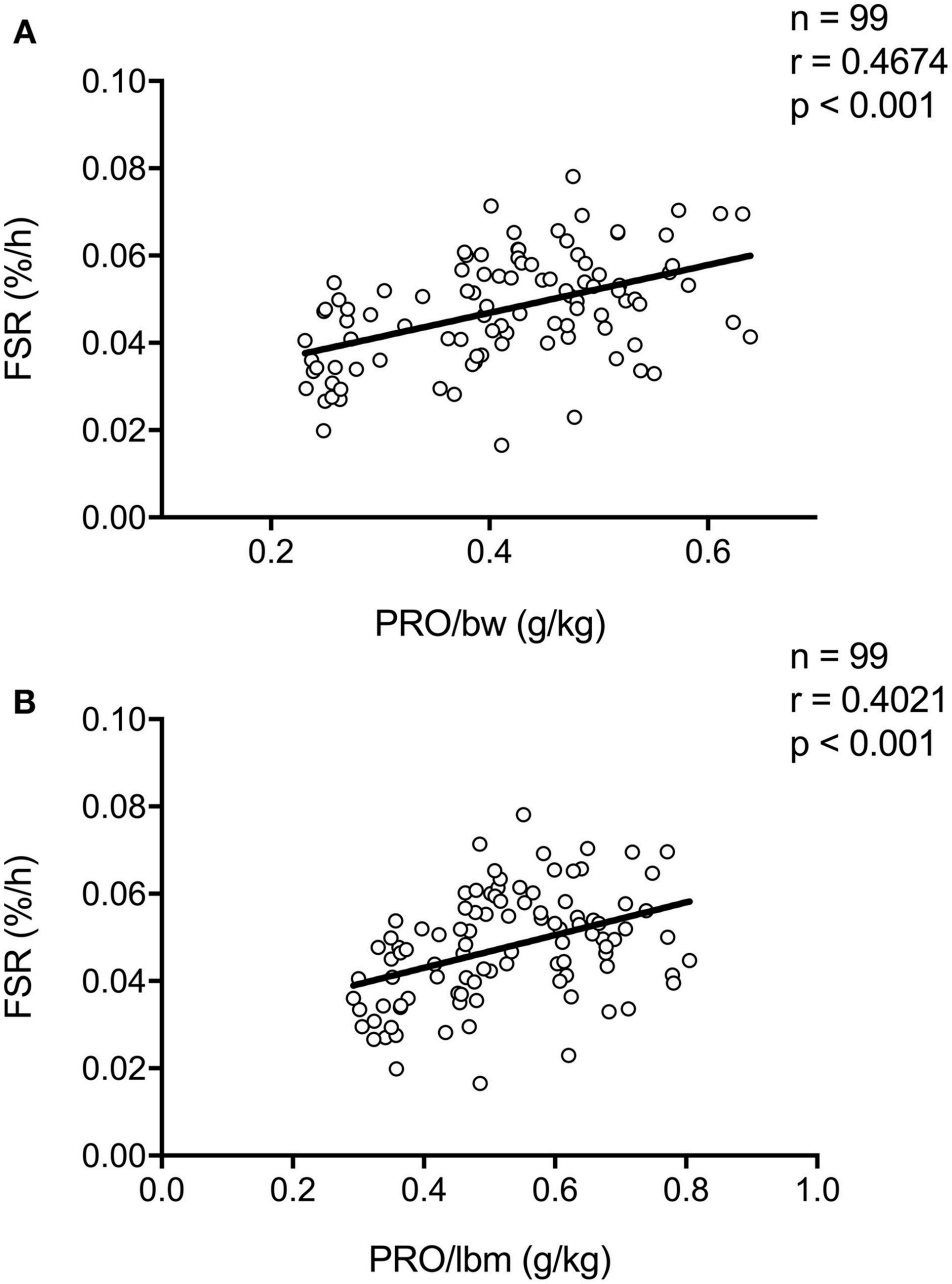
Scatter plot of the correlation between overnight (mixed muscle or myofibrillar) protein fractional synthetic rate (FSR) as calculated based intravenous infusion of L-[ring-^2^H_5_]_−_phenylalanine and **(A)** protein ingested per kilogram bodyweight (BW) or **(B)** protein ingested per kilogram lean body mass (LBM) in humans. Collapsed data set from previously published studies (11, 13, 14, 17, 18).

In all our work in which we assessed the impact of pre-sleep protein ingestion on overnight muscle protein synthesis, participants received a pre-specified absolute amount of protein to ingest prior to sleep. However, total daily protein intake recommendations such as the Recommended Dietary Allowance (RDA) are often expressed relative to bodyweight, i.e., in g/kg. More recently, per-meal protein recommendations have also been expressed relative to bodyweight and lean body mass (30). Thus far, only one study has directly investigated the impact of lean body mass on the muscle protein synthetic response to protein ingestion. Macnaughton et al. (31) assessed the impact of whey protein ingestion (20 or 40 g) on (day-time) post-exercise muscle protein synthesis between young adults with a relative low compared with a high amount of fat free mass (~59 vs. 77 kg fat free mass, respectively). Interestingly, no differences in post-exercise muscle protein synthesis rates after the ingestion of 20 or 40 g of whey protein were observed between groups. These data suggest that lean body mass is not a strong modulator of protein requirements during the first couple of hours of post-exercise recovery. To provide further insight into the relation between relative protein intake (g/kg body weight) and the subsequent postprandial response, we have collapsed our data sets on previously performed overnight muscle protein synthesis studies (*n* = 99). Here we show a positive association between protein intake (g/kg body weight, or g/kg lean body mass) and overnight muscle protein synthesis rates (Figures 2A,B). This positive association with relative protein intake (g/kg) was also observed when only analyzing data from subjects who did not perform prior resistance-type exercise (Supplemental Figure 1A, *n* = 56), and those that did perform prior exercise (Supplemental Figure 1B, *n* = 43). Furthermore, similar trends were observed for both young (Supplemental Figure 1C, *n* = 44), and older (Supplemental Figure 1D, *n* = 55) adults separately. We speculate that pre-sleep protein requirements may be personalized based on bodyweight or lean body mass, however, more direct experimental comparisons are warranted to further corroborate this speculation.

## Pre-Sleep Protein Ingestion and Appetite

A number of studies have recently assessed whether ingestion of a relative low-energy beverage prior to sleep could alter appetite, and/or cardio-metabolic risk factors the following morning in various populations. In a randomized, double-blind, cross-over study, Madzima et al. (32) compared the ingestion of various pre-sleep drinks containing different macronutrients (carbohydrate vs. protein) and different protein compositions (whey vs. casein vs. 50% blend) on satiety and resting energy expenditure on the following morning in healthy young men. In this study pre-sleep beverage consumption did not modulate satiety assessed the following morning (32). Interestingly, the authors did show that, irrespective of beverage composition, consumption of a pre-sleep caloric drink increased resting energy expenditure, without inhibiting fat oxidation, assessed the next morning when compared with a non-caloric placebo in healthy young men (32). These findings, together with an overnight increase in muscle protein synthesis rates following pre sleep protein ingestion (10, 11, 13, 14, 16–18), imply that protein ingestion prior to sleep does not appear to have negative effect on resting energy expenditure and fat metabolism the next morning and may be a viable option to support overnight muscle reconditioning and, as such, provide a competitive advantage to healthy young individuals/athletes. However, contrasting results have been reported by Kinsey et al. (33) applying the same research protocol and beverage composition in overweight and obese women. This study showed that the ingestion of a pre-sleep drink, irrespective of macronutrient composition, led to a greater subjective feeling of satiety the next morning. In addition, the morning following the nighttime ingestion of carbohydrate-only or protein-only pre-sleep beverages resulted in small but significant increases in resting insulin concentrations and subsequent an indicator of insulin resistance (assessed by Homeostatic Model Assessment of Insulin Resistance; HOMA-IR) (33). This indicates that in sedentary overweight and obese women ingestion of carbohydrate and/or protein beverages prior to sleep may elicit unfavorable metabolic effects. Interestingly though, the same research group has shown that these negative effects are completely abolished in overweight and obese subjects when protein and/or carbohydrate ingestion prior to sleep is combined with prolonged exercise training (34). But to truly assess whether the favorable (or absence of potential unfavorable) effects of pre-sleep protein ingestion *per se* on resting energy expenditure, glucose/fat metabolism and satiety the next morning, as observed in healthy young men (32), is also present in obese individuals, a comparison with a non-nutritive placebo would be prerequisite under well-standardized condition with regards to food intake during the day. Therefore, the same authors performed a cross-over follow-up study comparing pre-sleep protein ingestion (30 g protein) with a non-nutritive placebo in obese men during a period of well-controlled dietary intake. Here they showed that pre-sleep protein intake did not affect fat or glucose metabolism, resting energy expenditure, and did not suppress appetite the following morning in young obese men compared with a non-nutritive placebo. (35). Altogether, these studies indicate no direct negative effects on next morning appetite and resting energy metabolism when a low-energy, single nutrient/protein beverage is consumed prior to sleep. However, it is important to note that the impact of pre-sleep protein consumption on resting metabolic rate *during* overnight sleep remains to be established.

## Protein Intake Distribution

Distribution of protein intake over the day has been showed to be an important factor to maximize daily muscle protein synthesis rates and, as such, to optimize muscle reconditioning. A balanced distribution of daily protein intake over three main meals has been shown to result in higher 24 h muscle protein synthesis rates when compared with an unbalanced distribution, in which most protein is consumed at dinner (36). In support, a more balanced pattern of protein intake during 12 h of post-exercise recovery has been reported to result in higher muscle protein synthesis rates when compared with “*the ingestion of the same total amount of protein provided in less frequent but larger amounts (40 g every 6 h), or in more frequent, smaller amounts (20 g every 1.5 h)”* (37). Based on such findings, it is currently recommended to consume with each main meal at least 20 g protein, with no more than 4–5 h between meals to support muscle protein synthesis (8). However, ingestion of an additional post-exercise and/or pre-sleep protein meal may modulate the anabolic response to ingestion of the main meals. For example, previous work has suggested that a continuous supply of exogenous amino acids via intravenous infusion may actually blunt the initial rise in muscle protein synthesis rates (38). However, whether protein ingestion following exercise and/or prior to sleep influences the muscle protein synthetic response to the ingestion of subsequent protein-rich meals remains largely ambiguous. We have started to address this issue in a recent study by investigating whether protein ingestion immediately following exercise and before subsequent sleep could influence the muscle protein synthetic response to the consumption of a meal-like amount of protein the following morning (15). In this study, we demonstrated that the consumption of ample amounts of protein (60 g whey) before sleep did not alter post-prandial muscle protein synthesis rates to the following morning (15). In other words, these data suggest that the protein ingested during every meal signifies an distinctive opportunity to stimulate muscle protein synthesis and that subsequent rises in post-prandial muscle protein synthesis to each meal may be additive. This is relevant for the athletic population who usually consume more than 1.2 g protein kg bodyweight^−1^ day^−1^, with the majority of protein ingested during the three main meals, and only a small amount of protein ingested as an evening snack (39). Despite the relatively high amount of protein ingested earlier in the day, pre-sleep protein ingestion would presumably still provide an anabolic stimulus on overnight muscle protein synthesis rates, thereby enhancing daily muscle tissue re-conditioning (8).

## Long Term Effect of pre-Sleep Protein Ingestion

The more long-term effects of pre-sleep protein ingestion on skeletal muscle conditioning has also been assessed in healthy young men during a 12-week resistance type exercise training program. In this study we showed a greater increase in skeletal muscle mass and strength when participants ingested 27.5 g protein (50% casein + 50% casein hydrolysate) compared with a non-protein placebo prior to sleep (on both training as well as non-training days) during 12 weeks resistance type exercise training in healthy young men (12). These results provide evidence that ingesting a moderate amount (~30 g) of protein prior to sleep represents an effective intervention strategy to augment gains in skeletal muscle mass and strength during a resistance-type exercise training program in young men. However, it is important to note that in this study the pre-sleep protein supplementation was compared with a non-protein placebo, and not to protein supplementation at other time points. Whether pre-sleep protein ingestion has surplus benefits compared with protein supplemented at other time points throughout the day remains to be established. It has been hypothesized that pre-sleep protein supplementation increases the gains in muscle mass during prolonged exercise training mainly as a function of increased total protein intake, rather than by its specific timing of protein intake thereby improving protein intake distribution (8, 40). As meta-analyses data are required to clearly demonstrate that protein supplementation can augment gains in lean tissue mass and strength during prolonged resistance-type exercise training (41), it appears unlikely that a differential effect of protein supplement timing on the gains in muscle mass and/or strength can be detected in a longitudinal study design. In support, a recent study reported no statistical significant difference in fat-free mass gains when whey protein was supplemented in the evening when compared to protein supplemented in the morning (42). Nonetheless, the observed increase in fat-free mass was numerically greater when protein was ingested in the evening compared with the morning (+1.2 kg vs. +0.4 kg, respectively), leading the authors to speculate that the study might have been underpowered to significantly detect a relevant difference. In line, Joy et al. (43) observed no differences in muscle mass gains following 10 wks of resistance type exercise training between nighttime or daytime casein protein supplementation (35 g) in healthy young men. Again, however, no firm conclusion can be drawn from this study due to its limited sample size, as also acknowledged by the authors. To obtain insight in the required sample size for such work, we performed an *a posteriori* sample size calculation based on our previous work (12). With an alpha level set at 0.05 and with a power of 80%, it would require 24 healthy young volunteers in both the placebo and pre-sleep protein treatment to detect a hypertrophic effect of additional pre-sleep protein ingestion. Our calculations reveal that it would require substantially more young adults in both groups to detect a potential superior hypertrophic effect of pre-sleep protein ingestion compared to protein ingestion earlier in the day.

Although pre-sleep protein ingestion has been demonstrated to enhance the gains in skeletal muscle mass and strength during resistance-type exercise training in young men (12), this does not appear the case in older adults (44). In a recently published study, we reported no beneficial effect of protein ingestion (20 g whey + 1 g leucine) immediately after exercise and prior to sleep on the increase in skeletal muscle mass and strength during 12 weeks of resistance-type exercise training in healthy older men (44). Age-related factors, like habitual physical activity level, lower absolute workload, and the prevalence of anabolic resistance may explain the discrepant findings on the surplus effect of pre-sleep protein consumption on the muscle mass and strength gains following resistance exercise training between young and older adults. Overall, more sufficiently powered studies are warranted to investigate whether pre-sleep protein intake can augment gains in muscle mass and strength in response to long-term exercise training in general and it would be of great interest to assess its efficacy in specifically more clinically compromised older populations. Thus, far only one study has assessed and showed a positive impact of pre-sleep protein ingestion outside the laboratory (45). Results from this study suggest an accelerated recovery in the first days after a soccer match when protein was ingested prior to sleep following an evening soccer match in young adults (45). Clearly more research is required to further establish the true impact pre-sleep protein ingestion may have on muscle reconditioning and recovery in more applied setting of sports performance. Finally, little is known on the short as well as long-term effects of pre-sleep protein intake on subsequent muscle reconditioning in response to more intermittent and endurance-type exercise training.

## Conclusions

Protein ingested prior to sleep is effectively digested and absorbed during sleep, thereby increasing plasma amino acid availability and stimulating muscle protein synthesis during overnight sleep in both young and old. When pre-sleep protein intake is combined with exercise performed the same evening, overnight muscle protein synthesis rates will be further increased. Protein ingestion prior to sleep can be applied in combination with resistance type exercise training to further augment the gains in muscle mass and strength when compared to no protein supplementation. However, whether this beneficial effect on pre-sleep protein ingestion on muscle mass and strength gain during resistance-type exercise training are due to an increased total protein intake rather than by its specific timing remains elusive, and warrants further research. Protein ingestion before sleep has been hypothesized to represent an effective nutritional strategy to increase daily protein intake and, as such, to attenuate muscle mass loss in hospitalized older adults. In more clinically compromised older populations the combination with exercise or exercise mimetics (such as NMES) may further increase the efficacy of pre-sleep protein ingestion to improve overnight muscle protein balance.

## Author Contributions

TS, JT, and LvL drafted the manuscript. TS and JT prepared all the figures. IK, AH, and LV critically reviewed and revised the manuscript for important intellectual content. All authors contributed to manuscript revision, read, and approved the submitted version.

### Conflict of Interest Statement

Previous research work in our laboratory on pre-sleep protein ingestion has been supported by grants from GlaxoSmithKline and Top Institute Food and Nutrition. LvL, JT, IK, and LV have received research grants, consulting fees, speaking honoraria, or a combination of these, from Friesland Campina, Nutricia, and PepsiCo. The remaining authors declare that the research was conducted in the absence of any commercial or financial relationships that could be construed as a potential conflict of interest.

## Supplementary Material

Supplemental Figure 1 Pearson correlation between (mixed muscle or myofibrillar) overnight protein fractional synthetic rate (FSR) as calculated based upon intravenous infusion of L-[ring-^2^H_5_]_−_phenylalanine and protein consumption prior to sleep per kilogram bodyweight (BW) for **(A)** young and older adults in rest **(B)** young adults both in rest and following a single resistance exercise session **(C)** in young and older adults following a single resistance exercise session **(D)** older adults both in rest and following a single resistance exercise session. Collapsed data set from previously published studies (11, 13, 14, 17, 18).

## References

[1] McGlory C Phillips SM. Exercise and the regulation of skeletal muscle hypertrophy. Prog Mol Biol Transl Sci. (2015) 135:153–73. 10.1016/bs.pmbts.2015.06.018 26477914

[2] Biolo G Maggi SP Williams BD Tipton KD Wolfe RR. Increased rates of muscle protein turnover and amino acid transport after resistance exercise in humans. Am J Physiol. (1995) 268:E514–520. 10.1152/ajpendo.1995.268.3.E514 7900797

[3] Phillips SM Tipton KD Ferrando AA Wolfe RR. Resistance training reduces the acute exercise-induced increase in muscle protein turnover. Am J Physiol. (1999) 276:E118–24. 10.1152/ajpendo.1999.276.1.E118 9886957

[4] Burd NA Tang JE Moore DR Phillips SM. Exercise training and protein metabolism: influences of contraction, protein intake, and sex-based differences. J Appl Physiol. (2009) 106:1692–701. 10.1152/japplphysiol.91351.2008 19036897

[5] Wall BT Cermak NM van Loon LJ. Dietary protein considerations to support active aging. Sports Med. (2014) 44(Suppl. 2):S185–94. 10.1007/s40279-014-0258-7 25355192 PMC4213379

[6] Morton RW McGlory C Phillips SM. Nutritional interventions to augment resistance training-induced skeletal muscle hypertrophy. Front Physiol. (2015) 6:245. 10.3389/fphys.2015.00245 26388782 PMC4558471

[7] Wall BT Morton JP van Loon LJ. Strategies to maintain skeletal muscle mass in the injured athlete: nutritional considerations and exercise mimetics. Eur J Sport Sci. (2015) 15:53–62. 10.1080/17461391.2014.936326 25027662

[8] Trommelen J van Loon LJ. Pre-sleep protein ingestion to improve the skeletal muscle adaptive response to exercise training. Nutrients. (2016) 8:E763. 10.3390/nu8120763 27916799 PMC5188418

[9] Kinsey AW Ormsbee MJ. The health impact of nighttime eating: old and new perspectives. Nutrients. (2015) 7:2648–62. 10.3390/nu7042648 25859885 PMC4425165

[10] Groen BB Res PT Pennings B Hertle E Senden JM Saris WH. Intragastric protein administration stimulates overnight muscle protein synthesis in elderly men. Am J Physiol Endocrinol Metab. (2012) 302:E52–60. 10.1152/ajpendo.00321.2011 21917635

[11] Res PT Groen B Pennings B Beelen M Wallis GA Gijsen AP. Protein ingestion before sleep improves postexercise overnight recovery. Med Sci Sports Exerc. (2012) 44:1560–9. 10.1249/MSS.0b013e31824cc363 22330017

[12] Snijders T Res PT Smeets JS van Vliet S van Kranenburg J Maase K. Protein ingestion before sleep increases muscle mass and strength gains during prolonged resistance-type exercise training in healthy young men. J Nutr. (2015) 145:1178–84. 10.3945/jn.114.208371 25926415

[13] Holwerda AM Kouw IW Trommelen J Halson SL Wodzig WK Verdijk LB. Physical activity performed in the evening increases the overnight muscle protein synthetic response to presleep protein ingestion in older men. J Nutr. (2016) 146:1307–14. 10.3945/jn.116.230086 27281811

[14] Trommelen J Holwerda AM Kouw IW Langer H Halson SL Rollo I. Resistance exercise augments postprandial overnight muscle protein synthesis rates. Med Sci Sports Exerc. (2016) 48:2517–25. 10.1249/MSS.0000000000001045 27643743

[15] Wall BT Burd NA Franssen R Gorissen SH Snijders T Senden JM Presleep protein ingestion does not compromise the muscle protein synthetic response to protein ingested the following morning. Am J Physiol Endocrinol Metab. (2016) 311:E964–73. 10.1152/ajpendo.00325.2016 27780822

[16] Dirks ML Groen BB Franssen R van Kranenburg J van Loon LJ. Neuromuscular electrical stimulation prior to presleep protein feeding stimulates the use of protein-derived amino acids for overnight muscle protein synthesis. J Appl Physiol. (2017) 122:20–7. 10.1152/japplphysiol.00331.2016 27789768

[17] Kouw IW Holwerda AM Trommelen J Kramer IF Bastiaanse J Halson SL. Protein ingestion before sleep increases overnight muscle protein synthesis rates in healthy older men: a randomized controlled trial. J Nutr. (2017) 147:2252–61. 10.3945/jn.117.254532 28855419

[18] Trommelen J Kouw IWK Holwerda AM Snijders T Halson SL Rollo I Pre-sleep dietary protein-derived amino acids are incorporated in myofibrillar protein during post-exercise overnight recovery. Am J Physiol Endocrinol Metab. (2017) 273:2016 10.1152/ajpendo.00273.2016 28536184

[19] Beelen M Tieland M Gijsen AP Vandereyt H Kies AK Kuipers H. Coingestion of carbohydrate and protein hydrolysate stimulates muscle protein synthesis during exercise in young men, with no further increase during subsequent overnight recovery. J Nutr. (2008) 138:2198–204. 10.3945/jn.108.092924 18936219

[20] Moore DR Robinson MJ Fry JL Tang JE Glover EI Wilkinson SB. Ingested protein dose response of muscle and albumin protein synthesis after resistance exercise in young men. Am J Clin Nutr. (2009) 89:161–8. 10.3945/ajcn.2008.26401 19056590

[21] Witard OC Jackman SR Breen L Smith K Selby A Tipton KD. Myofibrillar muscle protein synthesis rates subsequent to a meal in response to increasing doses of whey protein at rest and after resistance exercise. Am J Clin Nutr. (2014) 99:86–95. 10.3945/ajcn.112.055517 24257722

[22] Katsanos CS Kobayashi H Sheffield-Moore M Aarsland A Wolfe RR. A high proportion of leucine is required for optimal stimulation of the rate of muscle protein synthesis by essential amino acids in the elderly. Am J Physiol Endocrinol Metab. (2006) 291:E381–387. 10.1152/ajpendo.00488.2005 16507602

[23] Rieu I Balage M Sornet C Giraudet C Pujos E Grizard J. Leucine supplementation improves muscle protein synthesis in elderly men independently of hyperaminoacidaemia. J Physiol. (2006) 575:305–15. 10.1113/jphysiol.2006.110742 16777941 PMC1819434

[24] Wall BT Hamer HM de Lange A Kiskini A Groen BB Senden JM. Leucine co-ingestion improves post-prandial muscle protein accretion in elderly men. Clin Nutr. (2013) 32:412–9. 10.1016/j.clnu.2012.09.002 23043721

[25] Pennings B Koopman R Beelen M Senden JM Saris WH van Loon LJ. Exercising before protein intake allows for greater use of dietary protein-derived amino acids for de novo muscle protein synthesis in both young and elderly men. Am J Clin Nutr. (2011) 93:322–31. 10.3945/ajcn.2010.29649 21084649

[26] Yang Y Breen L Burd NA Hector AJ Churchward-Venne TA Josse AR. Resistance exercise enhances myofibrillar protein synthesis with graded intakes of whey protein in older men. Br J Nutr. (2012) 108:1780–8. 10.1017/S0007114511007422 22313809

[27] Yang Y Churchward-Venne TA Burd NA Breen L Tarnopolsky MA Phillips SM. Myofibrillar protein synthesis following ingestion of soy protein isolate at rest and after resistance exercise in elderly men. Nutr Metab. (2012) 9:57. 10.1186/1743-7075-9-57 22698458 PMC3478988

[28] Wall BT Dirks ML van Loon LJ. Skeletal muscle atrophy during short-term disuse: implications for age-related sarcopenia. Ageing Res Rev. (2013) 12:898–906. 10.1016/j.arr.2013.07.003 23948422

[29] Wall BT Dirks ML Verdijk LB Snijders T Hansen D Vranckx P. Neuromuscular electrical stimulation increases muscle protein synthesis in elderly type 2 diabetic men. Am J Physiol Endocrinol Metab. (2012) 303:E614–23. 10.1152/ajpendo.00138.2012 22739107

[30] Moore DR Churchward-Venne TA Witard O Breen L Burd NA Tipton KD. Protein ingestion to stimulate myofibrillar protein synthesis requires greater relative protein intakes in healthy older versus younger men. J Gerontol A Biol Sci Med Sci. (2015) 70:57–62. 10.1093/gerona/glu103 25056502

[31] Macnaughton LS Wardle SL Witard OC McGlory C Hamilton DL Jeromson S. The response of muscle protein synthesis following whole-body resistance exercise is greater following 40 g than 20 g of ingested whey protein. Physiol Rep. (2016) 4:e12893. 10.14814/phy2.12893 27511985 PMC4985555

[32] Madzima TA Panton LB Fretti SK Kinsey AW Ormsbee MJ. Night-time consumption of protein or carbohydrate results in increased morning resting energy expenditure in active college-aged men. Br J Nutr. (2014) 111:71–7. 10.1017/S000711451300192x 23768612

[33] Kinsey AW Eddy WR Madzima TA Panton LB Arciero PJ Kim JS. Influence of night-time protein and carbohydrate intake on appetite and cardiometabolic risk in sedentary overweight and obese women. Br J Nutr. (2014) 112:320–7. 10.1017/S0007114514001068 24833598

[34] Ormsbee MJ Kinsey AW Eddy WR Madzima TA Arciero PJ Figueroa A The influence of nighttime feeding of carbohydrate or protein combined with exercise training on appetite and cardiometabolic risk in young obese women. Appl Physiol Nutr Metab. (2015) 40:37–45. 10.1139/apnm-2014-0256 25409324

[35] Kinsey AW Cappadona SR Panton LB Allman BR Contreras RJ Hickner RC. The effect of casein protein prior to sleep on fat metabolism in obese men. Nutrients. (2016) 8:E452. 10.3390/nu8080452 27472361 PMC4997367

[36] Mamerow MM Mettler JA English KL Casperson SL Arentson-Lantz E Sheffield-Moore M. Dietary protein distribution positively influences 24-h muscle protein synthesis in healthy adults. J Nutr. (2014) 144:876–80. 10.3945/jn.113.185280 24477298 PMC4018950

[37] Areta JL Burke LM Ross ML Camera DM West DW Broad EM. Timing and distribution of protein ingestion during prolonged recovery from resistance exercise alters myofibrillar protein synthesis. J Physiol. (2013) 591:2319–31. 10.1113/jphysiol.2012.244897 23459753 PMC3650697

[38] Bohe J Low JF Wolfe RR Rennie MJ. Latency and duration of stimulation of human muscle protein synthesis during continuous infusion of amino acids. J Physiol. (2001) 532:575–9. 10.1111/j.1469-7793.2001.0575f.x 11306673 PMC2278544

[39] Gillen JB Trommelen J Wardenaar FC Brinkmans NY Versteegen JJ Jonvik KL. Dietary protein intake and distribution patterns of well-trained dutch athletes. Int. J. Sport Nutr. Exerc. Metab. (2016) 27:105–14. 10.1123/ijsnem.2016-0154 27710150

[40] Reidy PT Rasmussen BB. Role of ingested amino acids and protein in the promotion of resistance exercise-induced muscle protein anabolism. J Nutr. (2016) 146:155–83. 10.3945/jn.114.203208 26764320 PMC4725426

[41] Cermak NM Res PT de Groot LC Saris WH van Loon LJ. Protein supplementation augments the adaptive response of skeletal muscle to resistance-type exercise training: a meta-analysis. Am J Clin Nutr. (2012) 96:1454–64. 10.3945/ajcn.112.037556 23134885

[42] Antonio J Ellerbroek A Peacock C Silver T. Casein protein supplementation in trained men and women: morning versus evening. Int J Exerc Sci. (2017) 10:479–86. 28515842 10.70252/QWHA8703 PMC5421981

[43] Joy JM Vogel RM Shane Broughton K Kudla U Kerr NY Davison JM. Daytime and nighttime casein supplements similarly increase muscle size and strength in response to resistance training earlier in the day: a preliminary investigation. J Int Soc Sports Nutr. (2018) 15:24. 10.1186/s12970-018-0228-9 29764464 PMC5952515

[44] Holwerda AM Overkamp M Paulussen KJM Smeets JSJ van Kranenburg J Backx EMP Protein supplementation after exercise and before sleep does not further augment muscle mass and strength gains during resistance exercise training in active older men. J Nutr. (2018) 148:1723–32. 10.1093/jn/nxy169 30247714

[45] Abbott W Brett A Cockburn E Clifford T Presleep casein protein ingestion: acceleration of functional recovery in professional soccer players. Int J Sports Physiol Perform. (2018) 11:1–24. 10.1123/ijspp.2018-0385 30204517

