# Supplementary figures and images for: The Impact of Pre-sleep Protein Ingestion on the Skeletal Muscle Adaptive Response to Exercise in Humans: An Update
Source: Front Nutr. 2019 Mar 6;6:17. doi: 10.3389/fnut.2019.00017 (PMC6415027; doi:10.3389/fnut.2019.00017)

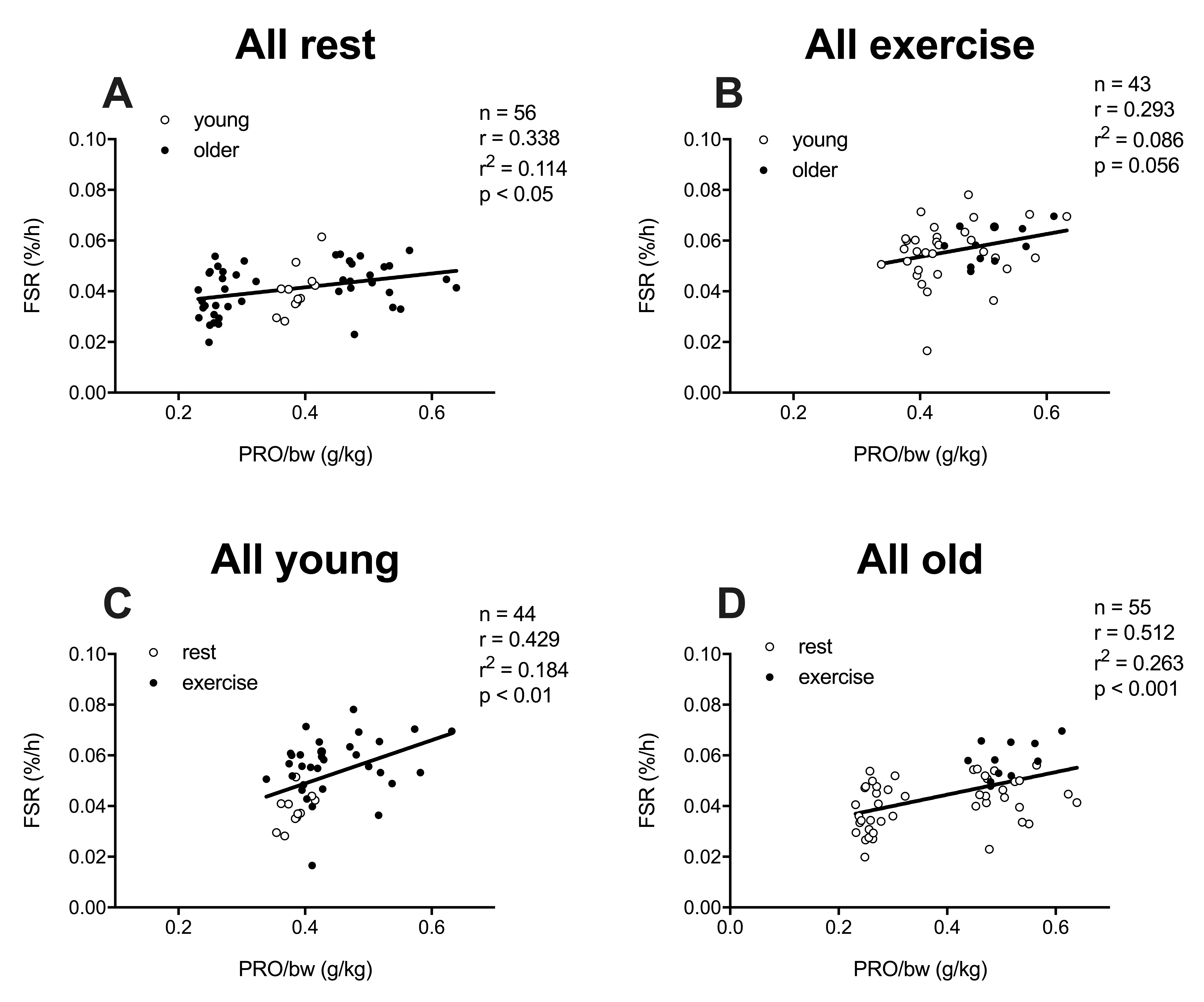

Supplement: Supplemental Figure 1 — Pearson correlation between (mixed muscle or myofibrillar) overnight protein fractional synthetic rate (FSR) as calculated based upon intravenous infusion of L-[ring-2H5]−phenylalanine and protein consumption prior to sleep per kilogram bodyweight (BW) for (A) young and older adults in rest (B) young adults both in rest and following a single resistance exercise session (C) in young and older adults following a single resistance exercise session (D) older adults both in rest and following a single resistance exercise session. Collapsed data set from previously published studies (11, 13, 14, 17, 18). [file Image_1.tiff]
